# Producer or Consumer? The House, the Garden and the Sourcing of Vegetables in Britain, 1930–1970

**DOI:** 10.1080/14780038.2019.1597414

**Published:** 2019-04-18

**Authors:** Sophie Greenway

**Affiliations:** Centre for the History of Medicine, University of Warwick, Coventry, UK

**Keywords:** Health, gardening, domesticity, gender, food

## Abstract

This article will ask why, given the discovery of vitamin C, Britain did not become a nation of domestic vegetable producers. It will explore how health concerns were central to domestic decision-making regarding the sourcing of vegetables in mid-twentieth-century Britain and will trace the ways in which the representation of women, as discerning consumers and guardians of the hygienic home, mitigated against the use of the domestic space of the garden for vegetable production. By the 1960s, the cleanliness of the home had extended into the garden, which was filled with labour-saving features to enhance al-fresco family life.

## Introduction

By examining relationships between the inside and outside, between house and garden, and between shopping and growing, this article provides a fresh perspective on the history of food choices and domestic practice in mid-twentieth-century Britain. In so doing, it draws on histories of domestic work, of hygiene and of modernism. Early twentieth-century understandings of hygiene combined ideas of wholesome regimen, originating in ancient Greece, with newer knowledge of germ theory.^1^ These concepts of hygiene were central to modernist design of both mass-produced household goods and domestic architecture.^2^ The interwar years saw a huge expansion in suburban living for both the middle and working class, accompanied by the emergence of a basic pattern of home and garden found in both private and municipal housing.^3^ An overlooked element of this social and cultural change is the entanglement of the new culture of clean suburban homes with decision-making about the garden. Although associations of fresh fruits and vegetables with health were never lost, this article will argue that the constant presence in women’s magazines of the idea that the housewife should maintain the sanctity of the hygienic domestic space, in particular against threats of dirt on the knees of boys and the boots of men, influenced domestic choices both inside and out. Associations between hygiene and domestic cleanliness shaped both shopping and gardening habits, altering the relationship of British people with the environment on their doorstep.

A focus on the period 1930–70 enables comparison of domestic practice in peacetime with that under the emergency conditions of the Second World War. By the 1930s, the incremental loss of vitamin C from harvested fruit and vegetables was well understood.^4^ The logical response to this new information would be to encourage local food growing to reduce the time from harvest to plate. The capacity of the British people to produce their own vegetables when it was thought necessary was demonstrated during the Dig for Victory campaign of the 1940s.^5^ Yet domestic vegetable production was not promoted as a means of securing the freshest food in peace time, before or after the war. Public debate about the combined problems of poverty, hunger and agricultural depression in the 1930s were framed in terms of a view of the public as consumers, and farmers as producers.^6^ This perspective was also present in discussions of nutrition and poverty in the journal *Public Health* during the mid-twentieth century, whilst government propaganda on public health regarding vegetables focused on the housewife’s role as a discerning consumer and cook.^7^

Today, home and community growing are seen as components of sustainable and healthy food systems, reducing food miles and leading to positive outcomes for both physical and mental well-being.^8^ An examination of domestic vegetable procurement in mid-twentieth-century Britain invites reflection on our choices in this regard. These choices are shaped not only by access to growing space or sources of fresh foods, but also by relationships and practices in the home, and by understandings of what it means to be healthy. Traditionally, women have been responsible for the cleanliness of the household and for producing meals. During the late nineteenth and early twentieth centuries, these responsibilities increased in significance as a result of new scientific understanding of the role of vitamins and germs in promoting and endangering health respectively. This article will argue that concern about hygiene, instead of encouraging women to procure vegetables from their own garden, contributed to the appeal of both purchased fresh vegetables, and canned produce marketed for “purity”. The role of hygiene in vegetable procurement was not something that was frequently discussed in mid-twentieth-century Britain, but a close examination of a range of women’s and gardening magazines will show how conflicting messages about fresh vegetables, at once providers of healthy vitamins and carriers of dirt and insects, were resolved through the portrayal of women as discerning consumers. Magazines presented women’s caring responsibilities, and particularly their policing of hygiene, as potentially problematic when seen in the context of vegetable growing. I will show how ambivalence about the role of women in the garden represents a significant and under-examined factor influencing mid-twentieth-century domestic practice.

This article will draw on four British magazines to explore the ways in which everyday domestic decision-making regarding vegetables was portrayed. I have examined textual and pictorial portrayals of domestic practice indoors and out in both articles and advertisements. Rachel Ritchie, Sue Hawkins *et al*. have shown how women’s magazines represent an under-researched element of twentieth-century print culture, in part because of their “composite nature”.^9^ This article uses the heterogeneity of magazine content to demonstrate the strength of cultural norms. No matter how diverse the articles and advertisements, they all show the housewife as responsible for the cleanliness of the home, and for the quality of the family’s food. Thus, as Ritchie *et al*. suggest, the varied nature of magazine content can be a benefit to historians, in this case illustrating a remarkable consistency of underlying messages about domestic practices.

The four magazines reviewed are *Woman’s Outlook, Home and Country, Amateur Gardening* and *House and Garden. Woman’s Outlook*, the monthly magazine published by the Women’s Co-operative Guild, was a left-wing magazine campaigning for the rights of women workers and promoting women’s involvement in civic life. Yet it also acted as a shop window for co-operative products, representing women as discerning consumers both of food and of household goods. It therefore portrays a variety of images of womanhood in mid-twentieth century Britain. *Home and Country*, the monthly magazine published by the National Federation of Women’s Institutes (NFWI), provides a largely rural perspective. The NFWI was apolitical and encouraged women to take part in civic life, by campaigning for both rural education and improvements in domestic conditions. The campaigning approach adopted by both *Woman’s Outlook* and *Home and Country* meant that they were keen to reflect the lived experience of readers: “how I enjoy my *Woman’s Outlook*. The recipes and hints and advice on lots of things, not the piffle we get in the many weekly’s”.^10^ Thus, these magazines can provide useful insights into the ways in which the homogenous ideal of domesticity was being lived. *Amateur Gardening* was selected as the Wartime Social Survey of 1942 identified it as the most popular gardening magazine.^11^ It was a weekly publication catering largely for the new residents of the expanding suburbs, many of whom were cultivating a garden for the first time.^12^ No gardening magazine for women existed in this period, but *Amateur Gardening* provides a useful comparison in terms of the portrayal of gender in a magazine aimed largely towards men. *House and Garden*, first published in 1936 as a supplement to *Vogue*, was targeted towards wealthy women readers who might have responsibility for a team of servants and for more than one home. It was the only magazine surveyed which ceased publication for the duration of the war, resuming in 1947.

The mid-twentieth century was a time in which a new mode of domesticity was constructed, as some moved into new suburban accommodation and others came to terms with life supported by fewer servants.^13^ The magazines reviewed reflected this domesticity both through aspirational advertising, and articles on household issues. In addition, readers wrote in with their experiences. I will use these heterogeneous sources, along with government reports, the journal *Public Health*, memoirs and advice books, to trace common assumptions about the house, the garden and the sourcing of vegetables. First, I will examine the ways in which class and rural or urban living impacted on domestic decision-making about vegetables, and how this changed over time. I will then examine the magazines’ representations of the responsibility of women for domestic hygiene and the family’s nutritional health. I will argue that these two factors contributed to decisions about vegetable procurement, especially given concurrent developments in food packaging and storage which contributed to the hygienic and nutritional appeal of shopping for vegetables. I will show how the garden was presented as a male preserve, and vegetable growing as an old-fashioned activity, one in which women were portrayed participating mainly in times of emergency, such as the Second World War. I will conclude by demonstrating that, during the post-war period, the garden was presented as an extension of the hygienic domestic realm, confirming the position of the modern British family as consumers, not producers, of vegetables.

### Class, the urban and the rural in domestic vegetable procurement

For some, a clean, modern, labour-saving home was unattainable due to the prohibitive cost of rent, furniture and commuting that a move to suburbia entailed.^14^ However, consumer surveys suggest that many purchased vacuum cleaners and washing machines in an effort to improve life in accommodation without running hot water or indoor toilets.^15^ Similar piecemeal change took place with respect to vegetable procurement during the mid-twentieth century. In 1930s Britain, choices about the production or consumption of vegetables were often restricted by a lack of money, time or growing space. Robert Roberts’ memoir of life in a Salford slum states that many of the poor ate few vegetables, not even potatoes.^16^ D. Caradog Jones’ Social Survey of Merseyside found that only 19% of Liverpool working-class families had gardens, and no mention was made of access to allotments.^17^ According to Bryan Magee, the poor in 1930s London would tour the markets at the end of the day to purchase fruit and vegetables unwanted by others, which would not have been in the freshest or cleanest possible state.^18^ *Home and Country* provides evidence that vegetable growing was common for working people in rural areas. The rural economy was very flexible, with bartering and co-operative growing combining with local ventures such as NFWI markets.^19^ However, one social survey of the 1930s pointed out that many of the rural poor could only access vegetables that they grew, meaning that their nutritional health fluctuated markedly between the seasons.^20^

Vegetables procured by scouring markets at the end of the day, or by growing where possible, would have required more involved preparation and waste disposal than those purchased from the shops. Picking off rotten or damaged parts of brussels sprouts, for example, or washing off mud and slugs all takes more time and produces more waste than heating up a can of peas, or indeed than preparing vegetables neatly trimmed by the greengrocer.^21^ Waste disposal also represented a burden of work for the mid-twentieth century housewife, as explained by a reader of *Home and Country* in the 1930s: “Somehow, we are always so very busy grappling with … getting our egg shells and tea leaves and fish bones disposed of, that we neglect to cultivate the arts of life.”^22^ Domestic practice in poorer urban and rural households in the 1930s was governed by need and involved either foods of poor quality or an intermittent supply. When these foods were available, their preparation placed an extra burden on the housewife.

The wealthy were unlikely to directly purchase or grow vegetables themselves, although this was not always clear when upper-class people wrote about domestic life. Within the pages of *House and Garden*, it is difficult to distinguish which practices were carried out by the mistress of the house, and which by servants. Judy Giles has written of the need felt by mistresses to understand the work of their servants, so that they could manage them effectively.^23^ Thus, a magazine might explain practical tasks that the reader would not carry out herself. In an article of 1939 describing how to organise a weekend party in one’s country cottage, the mistress sent shopping instructions to a “local cook” and could cater for unexpected guests using produce from the country garden.^24^ In addition to demonstrating the blurring of the urban and rural that was open to the wealthy, this evidence shows that the writer was not the gardener who provided fresh vegetables, despite phrases such as “You must, of course, grow a nucleus of greens”.

By relying on domestic servants, wealthy women could provide a varied diet, including fresh vegetables, for their families and guests during the 1930s. In this sense, they were consumers both of food from shops and of the services of gardeners and cooks. This experience of shopping and growing vicariously enabled magazines to include articles on domestic practices, which would be relevant both to those who managed servants, and those who had to carry out the tasks themselves, whether servants or housewives. This was especially useful in tactfully handling the reduction in the availability of paid domestic help. The wealthy could find mechanical solutions to their servant problem, whilst those lower down the social scale might dream of one day purchasing the foods and appliances that they saw in advertisements.^25^

In the 1930s, only wealthy women could afford servants to cook and clean for them, and gardeners to grow fresh vegetables. They could provide for their family’s health both nutritionally and hygienically without working hard or getting dirty themselves. Rising standards of living during the mid-twentieth century meant that many more people, by the 1960s, had access to purchased vegetables whether fresh, canned or as part of prepared foods such as soups.^26^ A significant component of this transition was the model of the housewife as consumer. The purchase of vegetables, whether fresh or processed, removed the need for someone in the household to grow them, in addition to the more involved work of washing, preparation and waste disposal.^27^ Thus, purchasing vegetables enabled women in lower social classes in 1960s Britain to provide for their family’s health with a significant reduction in the physical labour and contact with dirt that their mothers had experienced.^28^ Magazines were powerful vehicles for underlying messages about how this new life should be lived.^29^ In the following section, I will explore this phenomenon regarding two factors, hygiene and nutrition, that, I will argue, were significant in determining domestic decision-making regarding vegetable procurement.

### Hygiene, health and domestic space

**Figure 1.**
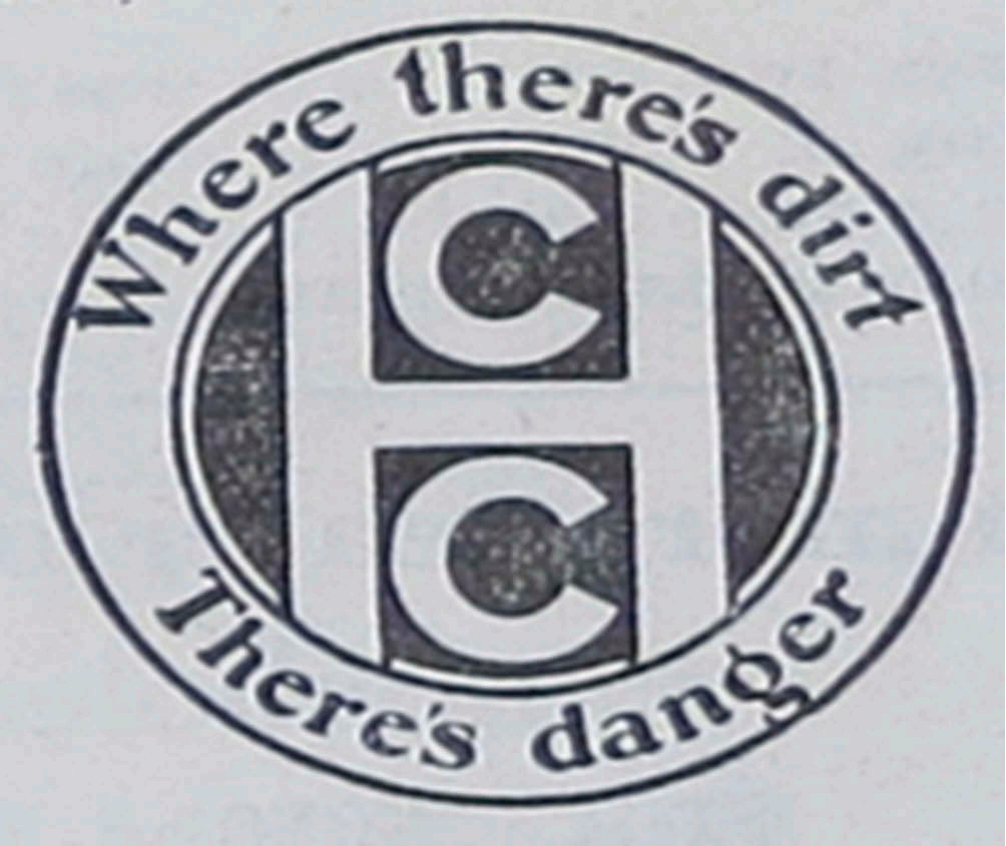
Logo of the Health and Cleanliness Council, The New Home, A Handbook for Tenants, back cover, Health and Cleanliness Council, 1937; Archive of the National Association of Teachers of Home Economics and Technology, earlier the Association of Teachers of Domestic Science, Modern Records Centre, University of Warwick (MSS.175/5/8/2).

Representations of vegetable growing were largely absent from magazine portrayals of peace-time normality in mid-twentieth-century Britain. My explanation for this absence hinges on the role of hygiene in framing perceptions of domestic space. Although health was linked to vegetables overtly and positively in terms of their vitamin content, this link co-existed with potential confusion over associations between muddy vegetables and the dirty soil which must be banished from the home.^30^ Nancy Tomes has shown how medical advice linking health to hygiene in the USA was propounded by both health reformers and manufacturers of household goods in the late nineteenth and early twentieth centuries. As a result, she argues, concern about infection became a key motivation in everyday domestic decision-making and practice.^31^ Such advice was also prominent in Britain, with the mid-nineteenth-century municipal reforms of Edwin Chadwick and Joseph Bazalgette paving the way for work focusing on individual responsibility in public health, such as the Health and Cleanliness Council (HCC), a voluntary organisation, which produced educational materials between the 1920s and 40s, with its motto “Where there’s dirt there’s danger” (see Figure 1).^32^ Tomes argues that the “germ panic” peaked in the USA around 1900, yet in Britain the linkage of hygiene and health was a common feature of both advice and advertisements during the first half of the twentieth century in women’s magazines particularly. The impact of this on women’s sense of domestic responsibility can be seen in letters from readers. A *Woman’s Outlook* letters page from 1950 contained advice on how to whitewash the house, a practice intended to reduce infection, whilst still providing treats for the children and glamour for the husband, so that he will not notice the disruption:

Try not to harass the family, and don’t do too much whitewashing at once, and so get irritable and nervy. Get some special biscuits for the kiddies’ tea, and don’t forget to put on a pretty apron (and some lipstick and powder) to greet hubby when he comes in; then he’ll be unmindful of the fact that he can’t find his slippers or his pipe or his paper or the dog’s lead or his seed catalogue. (Mrs.) G. Tonkin (Gillingham).^33^

**Figure 2.**
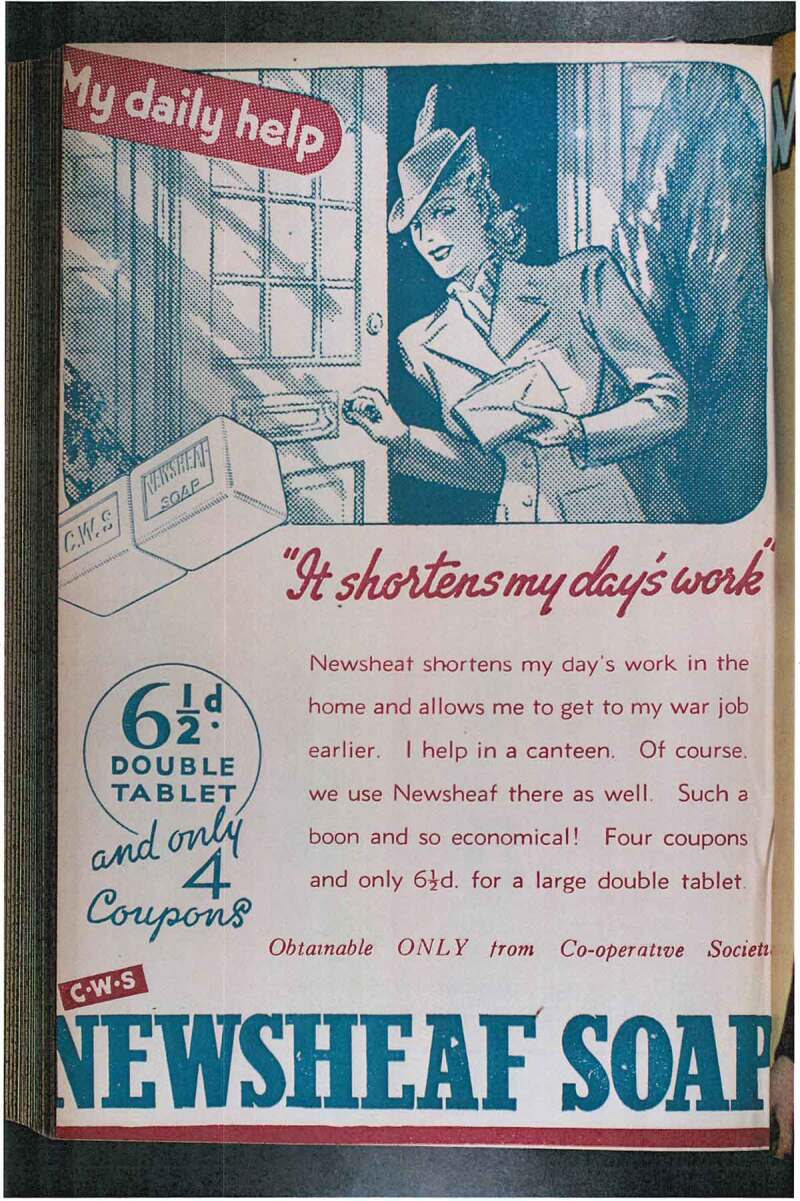
Woman’s Outlook, 9 October 1943, back cover, courtesy of Co-operative Press Ltd/www.thenews.coop.

**Figure 3.**
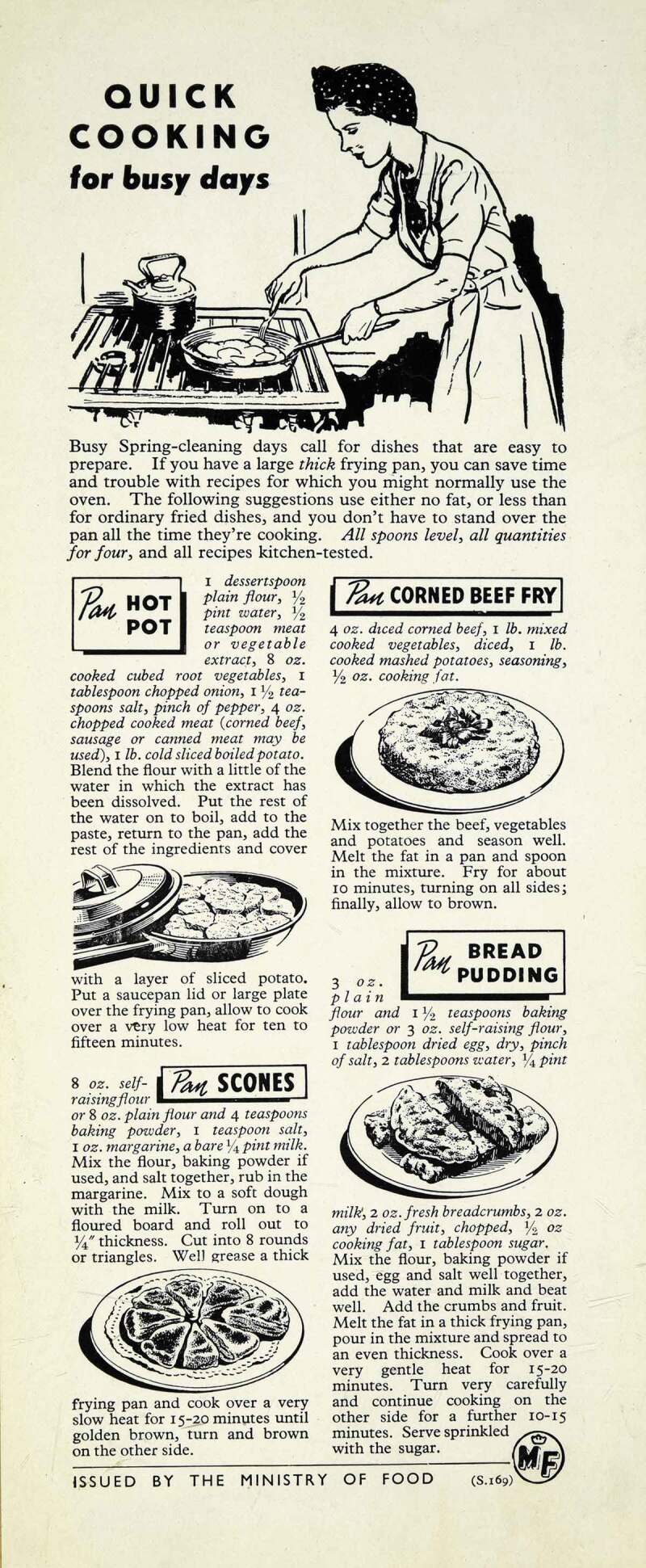
Ministry of Food, Album of Home and Women’s Magazine Advertising, 1945–8, MAF 223/22, courtesy of The National Archives.

**Figure 4.**
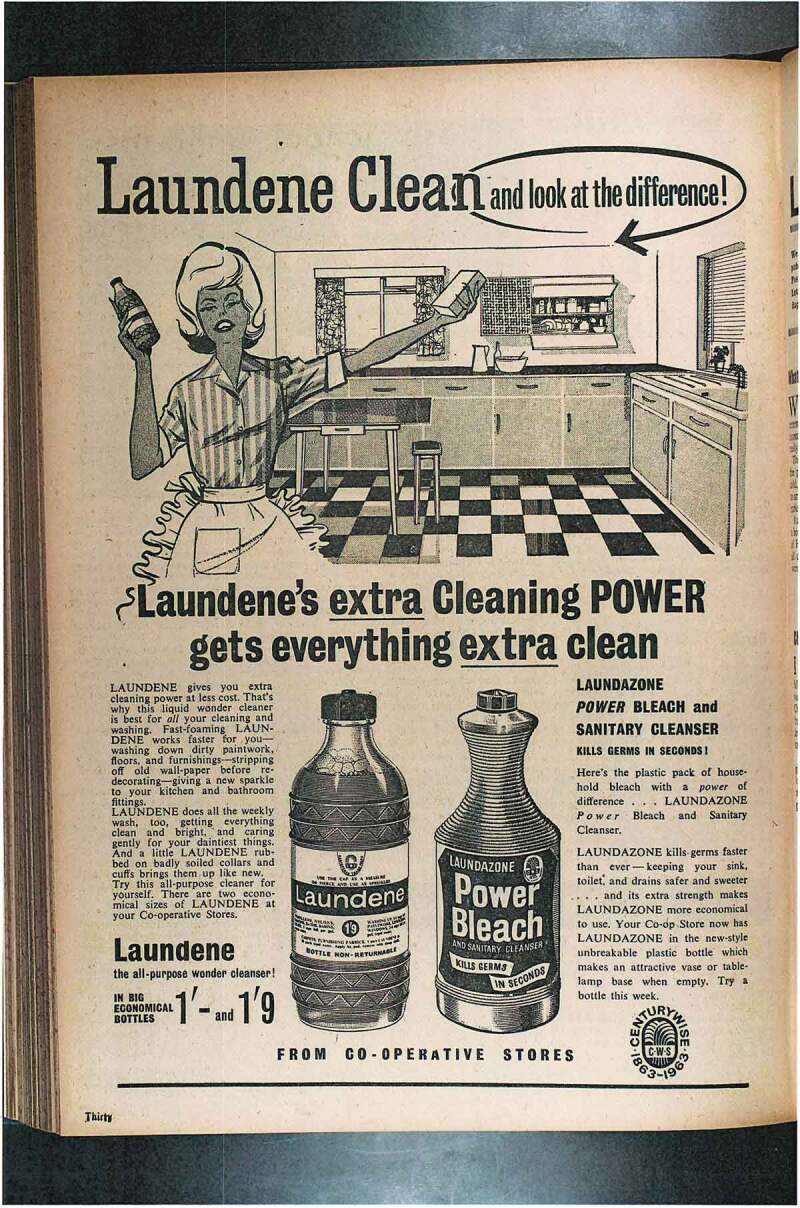
Woman’s Outlook, October 1963, 30, courtesy of Co-operative Press Ltd/www.thenews.coop.

Concern about hygiene played a significant role in the construction of the idea of domestic modernity that took place during the mid-twentieth century in Britain, with, as Ruth Schwarz-Cowan has shown, rising expectations of domestic cleanliness running in parallel with technological change.^34^ The association of domestic hygiene with femininity was emphasised particularly during the interwar period with the recognition by manufacturers and advertisers of the significance of female choice in consumption for the home.^35^ During the war, a Co-operative advertisement for soap showed a woman leaving for her “war job” having completed her domestic tasks, thanks to the soap, her “daily help” (see Figure 2).^36^ Thus, women’s caring role was shown as a constant, maintained, despite the war, with the assistance of Co-operative products. Government propaganda also emphasised this association of women with health, hygiene and the care of the home, with appeals to housewives to safeguard their family’s health through good cooking, especially when they were spring cleaning (see Figure 3).^37^ Angela Partington has shown how the association of women with domesticity was heightened in the post-war period of reconstruction, with the centrality of the family to the establishment of the welfare state, and the flooding of British markets with consumer goods in the early 1950s as wartime restrictions were lifted.^38^ Advertisements linked the idea of women’s responsibility for the home with hygiene and health. In 1960, a glamorous housewife, with a joyous sweep of the sponge, displayed her sparkling modern kitchen. The advertisement showed Co-operative Laundazone Power Bleach, which “kills germs faster than ever” (see Figure 4).^39^

**Figure 5.**
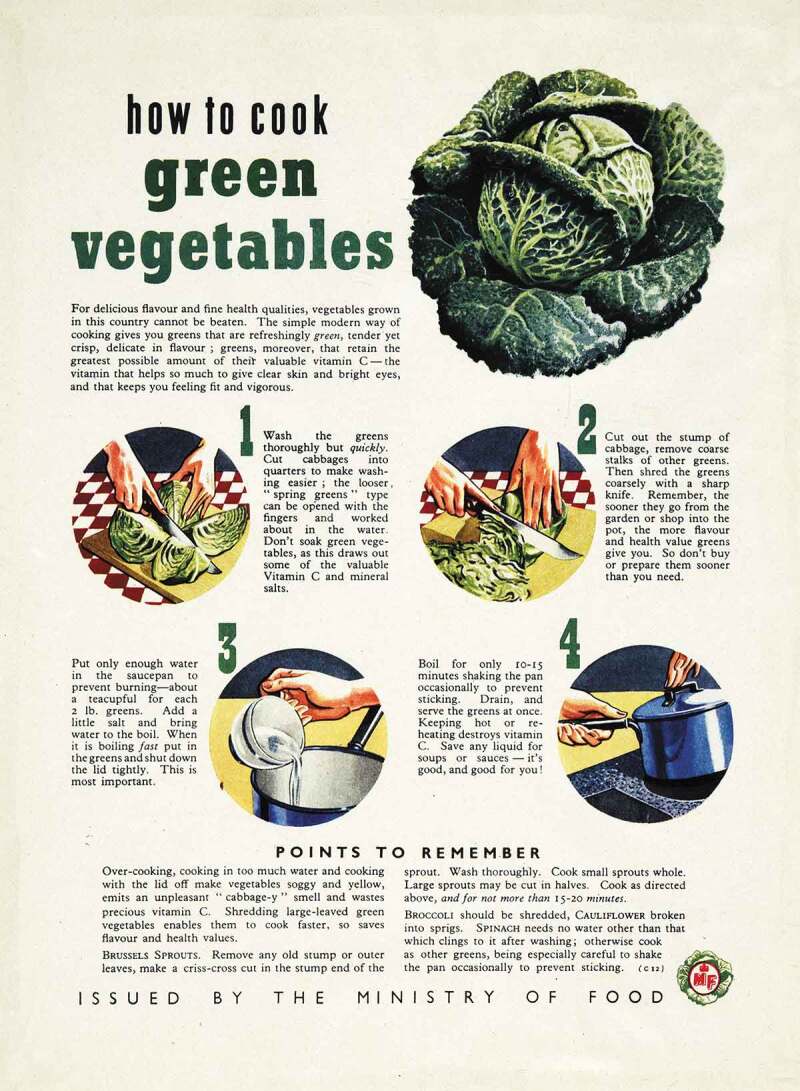
Ministry of Food ‘Home and Women’s Magazine Advertising’, 1945–8, MAF 223/22, courtesy of The National Archives.

Women were represented in *Woman’s Outlook* as responsible for the family’s nutritional health, as well as the cleanliness of the house. In the 1930s, the Co-operative movement cultivated the image of the woman with the basket, the discerning shopper who bolstered the family’s finances through the dividend. Articles and recipe pages also explained how to prepare healthy meals.^40^ In *Home and Country*, nutrition was discussed as the responsibility of the housewife: “I expect many a member who reads this article has already had experience in feeding her household on none too much money.”^41^ During the war, the theme of women’s responsibility for the family’s nutritional health was tackled by *Woman’s Outlook* in the regular column “The Shopping Basket”. In addition, numerous recipe pages explained how to preserve vitamins whilst cooking vegetables. *Home and Country* carried the Ministry of Food’s advice advertisements, which clearly showed housewives as responsible for the nutrition of the family.^42^ The Ministry of Food continued its propaganda throughout the late 1940s, fearing that “ignorance may well result in the paradox of greater food supplies but a less well-nourished nation” (see Figure 5).^43^ However, as rationing came to an end in the mid-1950s, living standards rose, and concern switched to malnutrition abroad and to diseases of affluence at home.^44^ *Woman’s Outlook* reflected these newly confident times, focusing on the convenience provided by modern food processing.^45^ An article on the dehydration of vegetables reassured readers that they were “very similar in nutritive value to the corresponding cooked fresh vegetable”.^46^ Thus, ideas of modernity, convenience and consumption were combined with reassurances regarding health.

Women’s twin responsibilities for hygiene and nutrition had the potential to cause confusion where vegetables were concerned. No-one recommended scrubbing a lettuce with carbolic, yet it had been in contact with soil and insects that were presented as risky to health. This tension was problematic for magazines too. Whilst recipes frequently advised washing vegetables thoroughly, health risks associated with soil were rarely discussed. The HCC’s handbook for new council house tenants published in 1938 attempted to strike a balance between promoting the health benefits of gardening and explaining the dangers present in soil: “Remember that what is good for the cabbages is not good for you. Digging your nails into a cake of soap before gardening will help to protect them from dirt.”^47^ Another HCC publication informed housewives that “Clean food is necessary for healthy life.” and “larder shelves should be scrubbed regularly with soap and hot water”, whilst recommending that vegetables be washed in plenty of water.^48^ This urging of the use of soap in all contexts except for vegetable preparation might have inclined housewives towards the purchase either of fresh vegetables, trimmed by the greengrocer or processed vegetables in packaging claiming their purity.

A further problematic issue was the impact on the domestic division of labour of vegetable growing in servantless households. All magazines studied avoided discussing this question, with one exception. A spin-off publication from *Amateur Gardening, The Woman’s Treasury for Home and Garden*, was published in 1936. This volume did not encourage women to garden but instead gave advice on the efficient processing of garden produce within the home. By promoting this book, a magazine which portrayed the garden as a male sphere was venturing into the female domain.^49^ The author, *Amateur Gardening* editor A.J. Macself, stated that the garden was “the concern and recreation of the man”, whilst “the wife is the one who puts the produce grown to good use for the pleasure of all”.^50^ Macself acknowledged that some women saw the garden as “a perpetual expense”, but felt that the wife was to blame: “if the products of a garden, … are utilised with discretion … it should prove to be in no sense an extravagance but a valuable asset in the management of the home”.^51^ Thus, if vegetables were grown in the garden, it was up to the wife to make good use of them. Most print media from mid-twentieth-century Britain that I have reviewed avoided this problem by portraying the procurement of vegetables for the family as a woman’s responsibility, which in peacetime was achieved through shopping.

It is my contention that, along with the appeal of modern, labour-saving products after the drudgery of the war years, concerns about hygiene underlay the transition experienced in British eating habits during the mid-twentieth century, as refrigerators, freezers and processed foods increased in popularity, and further that this concern about hygiene was intertwined with cultural conceptions of the safety of the home, and the role of women in ensuring that safety.^52^ Packaged and processed foods were marketed for their purity as well as their nutritional value and convenience, meaning that not only were the family protected from potentially harmful substances in the food, but that their preparation would leave little trace in the clean, and by implication hygienic, kitchen.^53^ *Woman’s Outlook* featured advertisements for “Ready Cooked Peas” which were prepared by boiling the whole can in water, as well as reports of tours of the Co-operative’s hygienic and modern canning factories, which used pure tin, “ideal for keeping foods pure and uncontaminated”.^54^ Thus, the family’s health would not be put at risk, but manufacturers were unable to claim that canned vegetables would provide the same level of vitamin C as fresh. In 1955, it was argued in *Woman’s Outlook* that canned produce was “preferable to fresh products of a poor quality or in an off-season” hardly a catchy advertising slogan.^55^ Thus, the advertising of processed foods, for which a consistent brand image could be constructed and greater profits made, shifted attention away from vegetables as a source of vitamin C, portraying them instead as a component of hygienically safe and convenient meals.^56^

Rural attitudes towards canning underline the distinction between urban consumer and rural producer. According to David Elliston Allen, reporting in 1968, canned vegetables were less popular in rural areas, yet during the war rural women were introduced to the practice of canning as part of the effort to preserve produce.^57^ Canning machines were sent from the USA and by October 1943 *Home and Country* reported that “Canning has definitely 'caught on'”.^58^ Canned foods were not always acceptable to urban women, however, with *Woman’s Outlook* in 1940 admitting that “Frequent use of a tin-opener has always been looked upon as … the mark of a lazy housewife.”^59^ This article sought to allay fears regarding both nutrition and hygiene, and recommended canned food as an excellent standby. Thus, the traditional practice of storing root vegetables in clamps and pickling others found a modern equivalent in canning. The consumption of canned foods, according to Elliston Allen, was more accepted in Birmingham than elsewhere, due to the greater proportion of housewives working outside the home.^60^ *House and Garden* advocated the use of “Carefully chosen tinned soups” bought at Selfridges as an essential component of hosting a country party.^61^ Although opinions of the merits of canned food varied regionally as well as by class, these accounts all agreed on the identity of their purchaser – the housewife. Presentation of this trope persisted throughout the mid-twentieth century as the opportunities for purchasing vegetables increased. Refrigeration facilitated the preservation of purchased fresh vegetables for longer periods, whilst freezers opened up the market for frozen peas in particular as an option needing little preparation and producing minimal waste.

### Domesticity and gender in the garden

Women were thus portrayed in print media as having responsibility for the health of the family, both in hygienic and nutritional terms. But what impact did these associations have on the portrayal of the garden? Vegetable growing was often associated with men, with the bucolic, the old fashioned, as well as with economic hardship.^62^ During the decade before the Second World War, numbers of allotments were boosted not because of the new knowledge of vitamins, but due to the Quakers’ campaign to provide unemployed men with a worthwhile pastime.^63^ Allotment holding was thus presented as a recourse in times of hardship, not a part of everyday life. Although anyone who was able was encouraged to Dig for Victory during the war years, information on vegetable growing in magazines decreased significantly after rationing ceased in 1954. A fashion for labour-saving gardening, which used modern materials such as concrete, was accompanied by a decline in the tenancy of allotments, prompting a government inquiry into their use in 1969.^64^ I contend that an overlooked factor in this idea of vegetable growing as a practice for times of desperation, not suited to the modern world, was the sheer dirtiness of it, and that the role of this factor can be detected by tracing the portrayal of women in the garden in print media.

During the 1930s, *Woman’s Outlook* did not discuss women gardening. There was no gardening page, and only occasionally were the gardens of readers mentioned. These references were to flower gardening as an example of good citizenship and community spirit.^65^ *Home and Country* contained evidence in readers’ letters and articles that rural women did garden, and did grow vegetables, but devoted little space during the 1930s to vegetable growing advice.^66^ *Amateur Gardening* included occasional references to lady gardeners, but largely conveyed the assumption that gardening was a hobby for men, and that those men were more interested in chrysanthemums than potatoes.^67^ It also perpetuated the view that the inside of the house was a gendered, separate sphere, in the way it portrayed the garden as a realm for men. Images in both advertisements and articles almost always featured men, unless they were demonstrating garden equipment as light or easy to use.^68^

**Figure 6.**
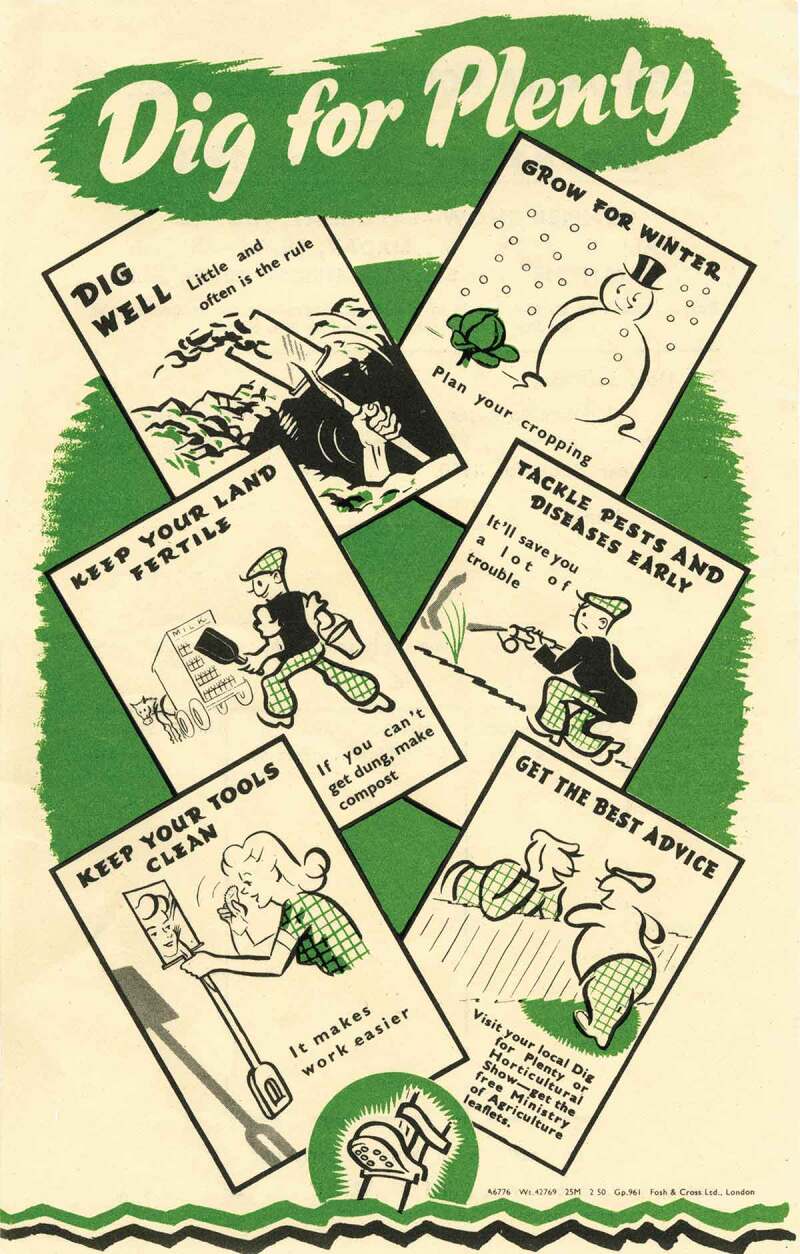
Ministry of Agriculture leaflet, late 1940s, courtesy of the Garden Museum.

The wartime campaign to encourage people to Dig for Victory was presented by the Government and the media as a temporary duty, necessitated by the emergency of war and the subsequent period of austerity.^69^ Ministry of Agriculture propaganda encouraged women to grow vegetables for the sake of their family’s health, assuring them that they could “get the older children to help”.^70^ In the late 1940s, a poster for the Dig for Plenty campaign, which encouraged people to continue growing vegetables during peacetime austerity, showed a man digging and collecting manure, whilst the woman keeps things clean, but actually checks her reflection (see Figure 6). *Woman’s Outlook, Home and Country* and *Amateur Gardening* all devoted space to vegetable growing during the war. *Woman’s Outlook* provides evidence of variation in the extent of home growing among readers. In 1942, whilst the unnamed author of the recipe page was able to state that: “There are few people now who have not got either garden or allotment, which provides vegetables of some sort all the year round”, Leonora Crossley’s assessment in her regular column was that working-class households were reliant on tinned vegetables for their Christmas dinner that year, due to high prices. Crossley did not mention the possibility that readers might grow their own.^71^ *Woman’s Outlook* showed images of women outside, but not actually gardening, and did not discuss the suitability of the practice for women.^72^

In January 1941, *Home and Country’s* editorial proposed that the NFWI’s contribution to the “national life and effort” would be “through the production and preservation of food”, and that “every village should aim at being self-supporting throughout the year in … vegetables.”^73^ By discussing the actions of “villages” as active entities, *Home and Country* delicately avoided issues of class and gardening work, since it was not clear which village members might be growing the produce. Local groups certainly responded, such as one in Cambridgeshire which grew extra onions to send to an institute in the North of England where the soil was less suitable for that crop.^74^ One account told of a group of women of indeterminate social class taking on an allotment. They were not familiar with vegetable growing but were pleasantly surprised by the results.^75^ *Amateur Gardening* featured a much higher proportion of articles on the garden as a site of production during the war, compared to the 1930s.^76^ The magazine was inconsistent in its portrayal of women in gardening, heading one editorial “Cropping plans: their significance to the family man”, whilst another referred to “able-bodied folk” as potential diggers for victory.^77^ An article on women’s gardening work as the war drew to a close explained that there would not be room for them in the trade, but they could continue gardening as a hobby.^78^ In these ways, both women gardening and vegetable growing were shown as necessary practices of war, not features of everyday life.

After the war, magazines reflected a preference for flower gardening, or some use of the garden clearly as a space for leisure, that was understandable after the constraints of war.^79^ Margaret Willes points out that women’s participation in gardening had increased since the war, and women’s magazines certainly reflected this, with *Woman’s Outlook* introducing a regular feature, “In the Woman’s Garden”. These articles were largely concerned with flower gardening.^80^ In 1945, one of *Woman’s Outlook’s* regular fiction articles featured a couple planning their new garden. The wife was keen that it should not be permanently devoted to vegetables: “Of course, we must be patriotic and grow more food now, but you will make something we can easily convert afterwards, won’t you, dear?”^81^ They planned to site a permanent vegetable patch behind a trellis at the bottom of the garden, and to temporarily grow additional vegetables in what would become herbaceous borders. The plot of the story revolved around the neighbour’s concerningly similar garden plan, as pegged out. However, the couple returned from their holiday to find that where they had assumed the neighbours would have trees and lawn, they had statues and crazy paving. The couple’s response was to copy their neighbours, ordering materials to construct a fountain in place of their planned apple tree.^82^ The story provides a rich picture of contemporary assumptions about gender, domesticity and the purpose of the garden. The husband was in control in the garden throughout, whilst the wife ensured his tea was ready promptly so there would be time for gardening in the evening. On return from holiday, the woman did not instantly join her husband in peering at the neighbours’ garden from their bedroom window, instead she checked what the butcher had delivered. Thus, provisioning for the house was clearly the woman’s responsibility, whilst decisions about the garden were taken by the man. They agreed that they wanted to grow mostly flowers after their “patriotic” duty was done. Food growing was a necessity, not a pleasure.^83^

*Home and Country* continued to present vegetable growing as one of many activities undertaken by the housewife, with a regular column on garden jobs appearing alongside one on household tasks, emphasising the traditional image of the frugal country housewife making the most of the produce available to her.^84^ During the period of post-war austerity, the magazine encouraged women to grow “standard” vegetables to supplement rations all year round.^85^ However, in the mid-1950s, gardening articles in *Home and Country* largely concentrated on flowers.^86^ By 1960, the magazine featured articles on flower arranging and garden history but not vegetable growing.^87^ This transition in emphasis towards flower gardening was accompanied by a shift in the magazine’s assessment of women’s physical capability. Whereas during the war *Home and Country* took for granted women’s ability to undertake heavy garden jobs, by 1955 the portrayal was very different.^88^ One article told of a visit to a rural cottage that the author remembered as having a very productive vegetable garden. She arrived to find the garden derelict, because her friend’s husband had been injured during the war. In discussing the need for a cultivating machine, the friend says: “I can’t do the really heavy work, but I can weed and plant and dress and sow”.^89^ Thus, while other reports of NFWI markets provide evidence that women’s production and selling of vegetables continued in rural areas, *Home and Country* also emphasised women’s relative physical weakness and, through its articles, encouraged them to grow flowers for the house as well as the garden.

**Figure 7.**
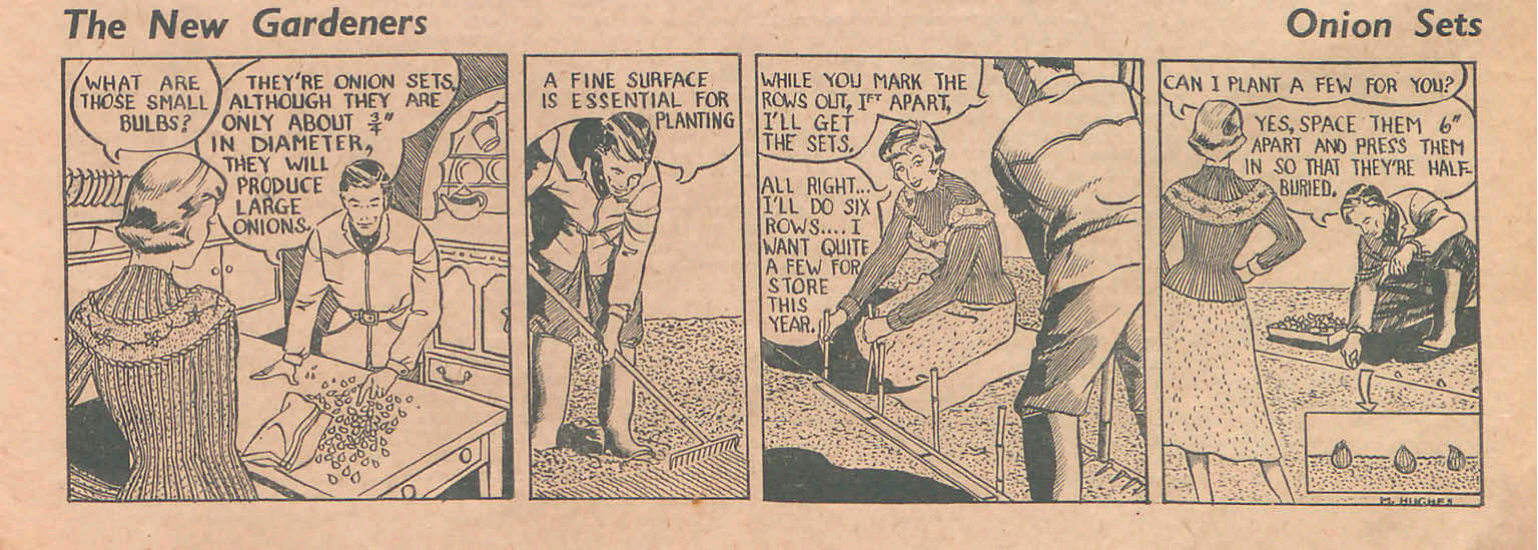
Amateur Gardening, 21 February 1957, 11, © British Library Board, 1957 General Reference Collection LOU.LON 124 [1957].

*Amateur Gardening* reverted to its portrayal of gardening as a hobby for men, a sentiment emphasised by the introduction of a separate page entitled “Specially for Women” giving advice on the indoor subjects of recipes and flower arranging.^90^ A cartoon strip published throughout 1957 entitled “The New Gardeners” at first glance seems to present an exception to the magazine’s traditional separation of roles (see Figure 7). A couple were shown carrying out a range of gardening tasks, including growing some vegetables. However, although the woman joined in with gardening activities, clear gender distinctions were maintained. The woman was always shown asking the man what to do, except where provisioning for the kitchen was concerned. She dressed glamorously. Her feet were hardly ever shown, but if they were, she wore feminine shoes and was pictured on a path or lawn. Any treading on earth was done by the man’s boot. If the woman helped plant bulbs, she sat on a mat.^91^ Thus, concerns about hygiene, about touching the earth, had become bound up in portrayals of gender and domesticity.

This visual portrayal of a woman actively gardening was rare in the post-war period. Whereas most print media in the late 1950s and 60s focused on the trend for labour-saving gardening, *Amateur Gardening* was aimed at those who practised horticulture as a hobby, and thus was more likely to show it. Meanwhile, throughout the mid-twentieth century, chemical companies marketed products associated with domestic hygiene, such as Izal and Jeyes Fluid, as solutions to garden pests and diseases. Izal, makers of medicated toilet paper, advertised their disinfectant for spraying against pests as well as soil sterilisation, claiming that the latter practice would “eradicate insect pests and the germs which cause soil sickness”.^92^ Gardeners were advised that “Stable or organic manures should be dug in before using Ster-Izal. If applied afterwards these might introduce new infection.”^93^ The garden was thus framed as a site in which to continue the battle against germs and infection that housewives had been waging since the beginning of the twentieth century. The maxim “Where there’s dirt there’s danger” was being applied to a living space in which the enemy was much harder to define than indoors, and in which the definition of dirt was less clear. Consumers were assured that through regular use of disinfectants, they would be protected as much outside as in.

The garden increasingly became seen as a site of leisure, with swimming pools, patios and ground cover planting arguably removing the need to touch the soil at all.^94^ Rising standards of living and increased foreign holidays meant people were interested in space in which to relax outside, rather than to practice gardening.^95^ John Brookes evoked the new fashion for eating al fresco with a cover image to his book *Room Outside* of a table laid for a meal set on a sunny patio. Inside the book, an elderly man was shown growing vegetables, whilst in other images younger adults relax on patios.^96^ A form of “labour-saving gardening” had been available to the wealthy throughout the mid-twentieth century, since their servants were the ones tending the gardens. The new labour-saving trend was pioneered in *House and Garden*, in which readers were assured that they could still enjoy their outside space even if their staff was depleted.^97^

## Conclusion

The modern era of tidy gardens as well as houses, as portrayed by the magazines and advice books, allowed little room for the messy practices of domestic food growing, let alone the entry of muddy vegetables into the gleaming kitchen. These publications cannot provide statistics on domestic vegetable production, but they do give a remarkably consistent impression of British domestic life. The mid-twentieth century saw continuity in the publications’ view of home vegetable growing, and especially vegetable growing by women, as a task undertaken out of necessity, either through poverty in the 1930s or due to war in the 1940s. Housewives, as the main decision-maker regarding food procurement, were reassured that modern retailing practices, along with better home storage, could provide for their family’s nutritional health.^98^ Once the post-war period of austerity was over, women were shown returning to their role as discerning consumer of food and household goods. Whilst they may have ventured into the garden, these tended to be tidy, sanitised spaces. The strength of the association between hygiene, health and consumption had served to blur the boundary between inside and outside by extending the domestic space out into the garden, encouraging British people to be consumers not only of vegetables, but of paving slabs and patio furniture.

Shoppers today find processed foods can be easier to access than fresh fruit and vegetables, especially those grown organically.^99^ In this sense, the twentieth-century association of muddy vegetables with poverty has been inverted. Substances applied to vegetables, such as chlorine on bagged salad, have, for some, come to represent a new form of dirt, whilst many appreciate the convenience of such products.^100^ By drawing attention to the significance of hygiene in portrayals of domesticity in mid-twentieth century Britain, I hope to provide a new way of thinking about what we buy, or grow, and why.

## Notes

1. Virginia Smith, *Clean: A History of Personal Hygiene and Purity* (Oxford, 2007), pp. 74–83.

2. Paul Overy, *Light, Air and Openness: Modern Architecture Between the Wars* (London, 2007), pp. 49–68.

3. Peter Scott, *The Making of the Modern British Home: The Suburban Semi and Family Life between the Wars* (Oxford, 2013), pp. 7–12; Smith, *Clean*, p. 313.

4. Dorothy Hollingsworth, ‘Developments Leading to Present-Day Nutritional Knowledge’, in Derek J. Oddy and Derek S. Miller (eds), *The Making of the Modern British Diet* (London, 1976), pp. 189–95.

5. Franklin Ginn, ‘Dig for Victory! New Histories of Wartime Gardening in Britain’, *Journal of Historical Geography*, 38.3 (2012), pp. 294–305.

6. John Boyd Orr, *Food, Health and Income; Report on a Survey of Adequacy of Diet in Relation to Income* (London, 1936). Boyd Orr made only occasional reference to ‘allotment output’ and ‘cottage produce’, p.s 54, 67.

7. *Public Health*, 62, p. 107; 71, p. 349.

8. Community Supported Agriculture, https://communitysupportedagriculture.org.uk/what-is-csa/ accessed 19 November 2018; David Buck, *Gardens and Health: Implications for Policy and Practice* (London, 2016).

9. Rachel Ritchie et al. (eds) *Women in Magazines: Research, Representation, Production and Consumption* (New York, 2016), p. 2.

10. *Woman’s Outlook (WO)*, August 1960, p. 40.

11. The National Archives (TNA), Wartime Social Survey, An Inquiry in to the Effects of the ‘Dig for Victory’ Campaign, 1942, RG 23/26, p. 29.

12. Scott, *Modern British Home*, p. 11.

13. Ibid., pp. 11–12; Ben Highmore, *The Great Indoors: At Home in the Modern British House* (London, 2014), p. 76; On managing without servants, see *House and Garden (HG)*, Spring 1949, p. 58.

14. Scott, *Modern British Home*, p. 9; Highmore, *Great Indoors*, pp. 84–8.

15. Miriam Glucksmann, *Cottons and Casuals: The Gendered Organisation of Labour in Time and Space* (Durham, 2000), p. 63; W.V. Hole and J.J. Attenburrow (eds) *Houses and People: A Review of User Studies at the Building Research Station* (London, 1966), p. 59.

16. Robert Roberts, *The Classic Slum: Salford Life in the First Quarter of the Century* (Manchester, 1971), p. 84.

17. University of Liverpool and D. Caradog Jones (eds), *Social Survey of Merseyside* (Liverpool, 1934), p. 117.

18. Bryan Magee, *Clouds of Glory: A Hoxton Childhood* (London, 2004), p. 59. See also *Public Health*, 49, p. 284.

19. *Home and Country (HC)*, March 1935, p. 157, May 1935, p. 249; W.J.Turner, *Exmoor Village* (London, 1947), p. 65.

20. *Public Health*, 52, pp. 358–62.

21. *WO*, 11 January 1947, p. 123.

22. *HC*, March 1935, p. 157.

23. Judy Giles, ‘Authority, dependence and power in accounts of twentieth-century domestic service’, in Lucy Delap, Ben Griffin, and Abigail Wills (eds), *The Politics of Domestic Authority in Britain since 1800* (Basingstoke, 2009), p. 210.

24. *HG*, 19 April 1939, p. 42.

25. *WO*, 15 November 1930, p. 41; 5 May 1945, p. 324. See also Highmore, *Great Indoors*, pp. 82–83, p. 250; Miriam Glucksmann, *Women Assemble: Women Workers and the New Industries in Inter-War Britain* (London, 1990), p. 239.

26. David Kynaston, *Family Britain, 1951–1957, Tales of a New Jerusalem* (London; New York, 2009), p. 663; Derek J. Oddy, *From Plain Fare to Fusion Food: British Diet from the 1890s to the 1990s* (Woodbridge, 2003), p. 171.

27. Fluctuating prices render comparison of the cost of canned versus fresh vegetables very difficult. See Charles Smith, *Britain’s Food Supplies in Peace and War: A Survey Prepared for the Fabian Society* (London, 1940), pp. 155–6.

28. Glucksmann, *Women Assemble*, p. 233.

29. Ryan, Deborah S., ‘“All the world and her husband”: The Daily Mail Ideal Home Exhibition, 1908–1939’, in Maggie Andrews and Mary M. Talbot (eds), *All the World and Her Husband: Women in Twentieth-Century Consumer Culture* (London, 2000), p. 18.

30. Health and Cleanliness Council (HCC), *Hints for the Busy Housewife*, 2nd edn (London, 1927), p. 33; *WO*, 1 March 1941, inside back cover; *AG*, 4 August 1942, p. 3.

31. Nancy Tomes, *The Gospel of Germs: Men, Women, and the Microbe in American Life* (Cambridge, MA; London, 1998), p. 17.

32. Anthony S. Wohl, *Endangered Lives: Public Health in Victorian Britain* (London, 1984); HCC, *Hints*.

33. *WO*, 15 April 1950, p. 176.

34. Ruth Schwartz Cowan, *More Work for Mother: The Ironies of Household Technology from the Open Hearth to the Microwave* (New York, 1983); Scott, *Modern British Home*, p. 12.

35. *Homes and Gardens (H&G)*, March 1930, p. ix; April 1930, p. lxxiv; *WO*, 8 March 1930, p. 309; Andrews and Talbot, *All the World*, p. 4.

36. *WO*, 9 October 1943, back cover.

37. TNA, Album of Home and Women’s Magazine Advertising, Ministry of Food advertisement feature, late 1940s, 1945–8, MAF 223/22.

38. Angela Partington, ‘The designer housewife in the 1950s’, in Judy Attfield and Pat Kirkham (eds), *A View from the Interior: Feminism, Women and Design* (London, 1989), p. 206.

39. *WO*, October 1963, p. 30. See also *H&G*, March 1959, p. 1; *HC*, November 1955, p. 426, December 1955, p. 460, July 1960, p. 252, January 1960, p. 32.

40. *WO*, 9 August 1930, pp. 650, 665.

41. *HC*, September 1930, p. 465. See also January 1935, p. 11, March 1935, p. 130.

42. Ibid., July 1940, p. 156, June 1942, p. 101.

43. TNA, Ministry of Food ‘Home and Women’s Magazine Advertising’, MAF 223/22.

44. Hollingsworth, ‘Developments’, p. 193.

45. *WO*, August 1966, p. 22.

46. Ibid., August 1960, p. 34–5. See also *H&G*, February 1959, p. 21.

47. HCC, *The New Home, A Handbook for Tenants* (London, 1938), p. 14.

48. HCC, *Hints*, p. 16.

49. Joanna Bourke, *Working-Class Cultures in Britain, 1890–1960: Gender, Class and Ethnicity* (London, 1994), pp. 67–70.

50. A. J. Macself, *The Woman’s Treasury for Home and Garden* (London, 1936), p. 17.

51. Ibid., p. 17.

52. Oddy, *From Plain Fare*, 169–88; Miriam Akhtar and Steve Humphries, *The Fifties and Sixties: A Lifestyle Revolution* (London, 2002), pp. 94–109; *HG*, April 1955, p. 99.

53. *WO*, 3 September 1939, inside front cover; *HC*, March 1935, pp. 155, 164; Oddy, *From Plain Fare*, p. 179.

54. *WO*, 3 September 1938, inside front cover; 27 April 1940, p. 816. See also *H&G*, February 1959, p. 150.

55. *WO*, 21 May 1955, p. 29.

56. ibid., 8 March 1930, back cover.

57. David Elliston Allen, *British Tastes: An Enquiry into the Likes and Dislikes of the Regional Consumer* (London, 1968), p. 155.

58. *HC*, October 1943, p. 151.

59. *WO*, 2 November 1940, p. 6.

60. Elliston Allen, *British Tastes*, p. 119.

61. *HG*, 19 April 1939, p. 42.

62. See image of a farmer on ‘Gaiety Grill’ range of tableware, produced by Crown Devon during the 1950s.

63. Lesley Acton, ‘Allotment Gardens: A reflection of history, heritage, community and self’, *Papers from the Institute of Archaeology* 21 (2011).

64. W.E. Shewell-Cooper, *Cut-Work Gardening* (Kingswood, 1963); Ideal Home, *Ideal Home Householders Guide*, 3 vols (London, 1966), I: Your Home and Garden, p. 174; Harry Thorpe, ‘The Homely Allotment, From Rural Dole to Urban Amenity: A Neglected Aspect of Urban Land Use’ *Geography* 60, 3 (July 1975), pp. 169–83.

65. *WO*, 25 January 1930, p. 205.

66. *HC*, March 1935, p. 157.

67. Margaret Willes, *Gardens of the British Working Class* (London and New Haven,2015), pp. 327–28. *AG*, 7 March 1936, p. 23.

68. *AG*, 6 August 1960, p. 19.

69. *AG*, 23 September 1939, p. 377.

70. Jennifer Davies, *Wartime Kitchen and Garden* (London, 1993), p. 78.

71. *WO*, 5 December 1942, p. 56; 19 December 1942, p. 66.

72. Ibid., 9 March 1940, p. 582; 7 November 1942, p. 16.

73. *HC*, January 1941, p. 3.

74. Ibid., January 1943, p. 2.

75. *HC*, September 1940, p. 187.

76. *AG*, 7 March 1936, 8 March 1941, 5 March 1946 and 7 March 1957.

77. Ibid., 10 August 1943, front cover; 2 March 1943, p. 7.

78. Ibid., 22 February 1944, p. 9.

79. Ibid., 11 September 1945, p. 7; *WO*, 27 January 1945, pp. 148–50.

80. Willes, *Gardens*, p. 348; *WO*, 12 March 1955, p. 31; 9 March 1957, p. 31; August 1960, p. 9; March 1966, p. 29.

81. *WO*, 27 January 1945, p. 148.

82. Ibid., 27 January 1945, pp. 148–50.

83. See also *HC*, July 1946, p. 119.

84. Ibid., June 1946, p. 99.

85. Ibid., December 1946, p. 203; July 1946, p. 119.

86. Ibid., September 1955, p. 349.

87. Ibid., September 1960, p. 311; October 1960, p. 376; September 1960, p. 337; July 1960, p. 236.

88. Ibid., December 1946, p. 203.

89. Ibid., December 1955, p. 443.

90. *AG*, 6 August 1960, p. 7.

91. Ibid., 3 January 1957, p. 11; 2 May 1957, p. 12; 8 August 1957, p. 13.

92. Sheffield Industrial Museums Trust, ‘Leaflet regarding the usage of Izal in the garden’, undated, 1950s or 1960s, MNNC0438. See also *HG*, June 1955, p. 94 on pesticides and herbicides as labour-saving.

93. Sheffield Industrial Museums Trust, ‘Label advertising Sterizal, for garden hygiene’, MNNC1030, undated, 1950s or 1960s.

94. Fred Streeter, *Labour-Saving Gardening* (London, 1956); Cement and Concrete Association, *Concrete in Garden-Making* (London, 1960); *HG*, Feb 1960, p. 66; Jane Brown, *The Pursuit of Paradise: A Social History of Gardens and Gardening* (London, 1999), pp. 267–68.

95. *HG*, June July 1950, p. 51; *H&G*, February 1959, p. 73.

96. John Brookes, *Room Outside* (London, 1969), p. 25.

97. *HG*, Spring 1949, p. 58, June 1955, p. 94, December 1955, p. 69.

98. Magnus Pyke, *Townsman’s Food*, (London, 1952), pp. 170–71.

99. ‘More than a million UK residents live in “food deserts” says study’, *Guardian*, 12 October 2018, https://www.theguardian.com/society/2018/oct/12/more-than-a-million-uk-residents-live-in-food-deserts-says-study, accessed 19 November 2018.

100. ‘Pre-washed salad is a dirty trick’, *Guardian*, 3 September 2008, https://www.theguardian.com/commentisfree/2008/sep/03/foodsafety.fooddrinks, accessed 8 September 2017.

